# Excess fatty acids induce pancreatic acinar cell pyroptosis through macrophage M1 polarization

**DOI:** 10.1186/s12876-022-02146-8

**Published:** 2022-02-19

**Authors:** Wenwen Xia, Zhaomin Lu, Wei Chen, Jianjun Zhou, Yan Zhao

**Affiliations:** 1 Department of Gastroenterology, Shanghai Tenth People’s Hospital of Tongji University, 301 Middle Yanchang Road, Jing’an, Shanghai, 200072 China; 2 Department of Gastroenterology, Zhangjiagang Second People’s Hospital, Zhangjiagang, Jiangsu 215633 China; 3 Research Center for Translational Medicine, Cancer Stem Cell Institute, East Hospital, Tongji University School of Medicine, 150 Jimo Road, Pudong, Shanghai 200135 China

**Keywords:** Free fatty acid, Cathepsin S, Acinar cell, Pyroptosis, Hyperlipidemia, Pancreatitis

## Abstract

Free fatty acid derived from hyperlipidemia contributes to the development of inflammation in the pancreas. Here we explore the molecular mechanisms of fatty acid-induced pancreatitis through cellular experiments and the construction of a mouse model of hyperlipidemic pancreatitis. We found that palmitic acid stimulation leads to M1 polarization of macrophage, which secretes cathepsin S via exosomes to pancreatic acinar cells and leads to activation of the caspase1-mediated classical pyrolysis pathway, resulting in inflammation and pancreatic tissue damage. In vivo experiments have also demonstrated that the high levels of fatty acids induced by hyperlipidaemia exacerbate the development of pancreatitis, and that cathepsin S inhibitors significantly alleviate hyperlipidemic pancreatitis. Therefore, cathepsin S may be a new target for the clinical treatment of hyperlipidemic pancreatitis.

**Supplementary Information:**

The online version contains supplementary material available at 10.1186/s12876-022-02146-8.

## Background

Hyperlipidemia is an elevation of lipids in the blood that gives rise to several diseases and is a rare but confirmed etiology of acute pancreatitis (AP) [1–4]. AP is a serious condition that can rapidly escalate to multiple organ dysfunction and subsequent mortality [5, 6]. Although alcohol abuse and gallstones are the most frequent cause of AP, triglyceride (TG) levels above 1000 mg/dL are also associated with the condition and can exacerbate alcohol-induced pancreatitis [7, 8]. Moreover, elevated TG-levels in AP are related to a worse prognosis in terms of mortality [9]. The high levels of TG associated with hyperlipidemia are hydrolyzed to generate free fatty acids (FFA). In particular, palmitic acid (PA), the most abundant FFA in the human body [10], is associated with the activation of various protein kinases, endoplasmic reticulum stress, increased reactive oxygen species (ROS) generation, and the recruitment of macrophage [11, 12]. The combination of these factors results in an inflammasome-related pancreatic cell death associated with caspase-1 and gasdermin D (GSDMD) known as pyroptosis [13–15]. However, the exact mechanism associating FFA with the pro-inflammatory response in the pancreas is unclear.

The emergence of AP involves components of the inflammatory response, which include acinar cells and macrophage [16, 17]. The response is triggered by the activation of digestive enzymes, such as trypsinogen, and the subsequent autodigestion of the pancreas, leading to necrosis, hemorrhage, and the degeneration of pancreatic parenchyma [6, 18]. M1 macrophage are involved in the initial response to pancreatic injury through the secretion and induction of pro-inflammatory cytokines and enzymes, such as TNFα, IL1β, and IL6 [19, 20]. The transcription factor interferon regulatory factor 5 (IRF5) is thought to be involved in the polarization of macrophage in response to inflammation and high expression is associated with M1 macrophage [21]. The genetic interference of IRF5 was found to reverse the AP-induced activation of the M1 phenotype to an M2 phenotype [17]. M2 are alternative macrophage that generate anti-inflammatory cytokines and are involved in the repair and remodeling of tissue [22, 23].

Cathepsins control the activity of trypsinogen in acinar cells and are involved in the initiation of AP [24, 25]. Levels of cathepsin B (CTSB), L (CTSL), and S (CTSS) are elevated in murine and human forms of AP [25]. CTSB is believed to be principally involved in the activation of trypsinogen because its inhibition or deletion attenuates inflammation and lowers the level of trypsinogen activity [26]. CTSL is involved in the degradation of trypsinogen, which would imply that it may have a role in alleviating AP [27]. However, the involvement of CTSL in AP may be more complex because its deletion attenuates inflammation. In AP, CTSS is found in macrophage whereas CTSB and CTSL are located predominantly in acinar cells [25]. The function of CTSS in AP is unclear but it is believed to participate in the induction of inflammation and its levels are increased in obesity [25, 28, 29].

In this study, we investigated the role of CTSS in FFA-stimulated macrophage-induced pancreatic acinar cell pyroptosis in hyperlipidemic pancreatitis (HP). We investigate the interaction of CTSS with IRF5 and whether the inhibition of CTSS can influence the progression of caspase-1 and GSDM-related pyroptosis in PA-stimulated acinar cells and in an HP mouse model.

## Methods

### Antibodies and reagents

Double-antibody (Procell Life Science & Technology, Wuhan, China; PB180120); anti-CTSS (Abcam, Cambridge, UK; ab134157); anti-IRF5 (Abcam, ab181553); anti-GSDMD (Abcam, ab219800); anti-trypsinogen (Abcam, ab166898); anti-CD11c (BD, Franklin Lakes, NJ, USA; 562454); anti-CD206 (Invitrogen, Carlsbad, CA, USA;17-2069-41); anti-CD63 (Invitrogen, 12-0639-41); anti-TSG101 (Invitrogen, MA1-23296); anti-ALIX (Abcam, ab225555); anti-caspase1 (Invitrogen, PA5-17570); anti-caspase-8 (Abcam, ab25901); anti-IL-1β (Abcam, ab2105); anti-ASC (Abcam, ab155970); anti-β-Actin (Abcam, ab8226).

Trypsase (Procell, PB180225); Ham’s F-12K medium (Procell, PM150910); high-sugar Dulbecco’s modified Eagle’s medium (DMEM; Procell, PM150210); Fetal bovine serum (FBS, Procell, 164210-500); polylysine (Procell, PB180523); phosphate-buffered saline (PBS, Procell, PB180327); DMSO (Sigma-Aldrich, St. Louis, MO, USA); CTSS ELISA kit (Abcam, ab155427); IL-1β, TNF-α,IL-10 ELISA kit (Sangon Biotech, Shanghai, China; D711047, D711114, D711045); Amylase, lipase ELISA kit (Sangon Biotech, D799323, D799801); Hematoxylin and eosin (HE) stain kit (Solarbio, Beijing, China; G1120-10); Trizol (Invitrogen); Tween-20 (Sinopharm, Beijing, China); Formaldehyde (Sinopharm); Lipofectamine™ RNAiMAX Transfection Reagent (13778075, Invitrogen); RT-RNA PCR Kit (Takara, Kyoto, Japan); Taqman Universal Master Mix II (Cat. No: 4440040, Thermo Fisher, Waltham, MA, USA); ExoQuick-TC (SBI, Palo Alto, CA, USA); T4 DNA ligase (Takara); Lipofectamine 3000 (Invitrogen).

### Cells and treatment

Rat pancreatic acinar cell lines (AR42J) were purchased from the American type culture collection (ATCC, Rockville, MD, USA; ATCC CRL1492). Cells were maintained in Ham’s F-12K complete growth medium with 20% FBS, at 37 °C with 5% CO_2_. The culture medium was renewed every 2–3 days. Murine macrophage cells were isolated from mice peritoneum. The mice (6–8 weeks old) were purchase from Shanghai Lab Animal Research Center (Shanghai, China). The cell isolation and culture steps were as follows. The mice were killed by cervical dislocation; 5–8 mL of high-sugar DMEM was aspirated with a sterile syringe, injected into the abdominal cavity, and gently massaged for 10 min. The abdominal cavity was opened; pale yellow abdominal fluid was aspirated with a syringe then placed in a centrifuge tube, and centrifuged at 400 × *g* for 10 min. The supernatant was discarded and the cellular precipitate was retained. Cells were resuspended in complete culture medium with mouse abdominal macrophage and inoculated in a culture dish at 37 °C, 5% CO_2_ at room temperature. The solution was changed after 24 h.

### Clinical specimens

Peripheral blood was collected from 64 patients with HP at the Tenth People’s Hospital of Shanghai, China. Blood samples were obtained from 57 AP patients and 7 normalsubjects. The blood was obtained without any pretreatment by using a vacuum blood tube. Then stored overnight at 2–4 °C and centrifuged at 3,600 × *g* for 15 min, the supernatant was collected and placed in liquid nitrogen and the precipitation was discarded. All patients signed informed consent and the study was approved by the Institute Research Ethics Committee of the Tenth People’s Hospital of Shanghai. The study methodologies conformed to the standards set by the Declaration of Helsinki.

### Animal model of hyperlipidemia pancreatitis

Twenty-four (6–8 weeks, 32 ± 2 g) C57BL/6J mice were purchased from the Shanghai Lab Animal Research Center (Shanghai, China). The mice were randomly housed in steel cages in a pathogen-free room with a 12 h light/12 h dark cycle and fed with a standard diet. All animal studies followed the Guide for the Care and Use of Laboratory The study methodologies conformed to the standards set by the Declaration of Helsinki.

After 1–2 weeks of acclimation, the mice were randomly divided into four groups with six individuals per group. One group was chosen as a blank control (Normal) and the mice were fed a standard chow diet throughout the whole experiment. The other three groups of mice were induced withhyperlipidemia by hyperlipidemia inducer Poloxamer 407 (P-407, Pluronic® F-127, P2443, Sigma-Aldrich) administration. P-407 was dissolved in PBS (pH = 7.4) and stored at 4℃ overnight. For long-term hyperlipidemia induction, 4 weeks of consecutive dosing (300 mg/kg, intraperitoneal injection) was administered. AP was induced after hyperlipidemia induction with injection of 5% taurocholic acid sodium (145-42-6, Solarbio, Beijing, China). Briefly, mice fast 12 h (free water) before anesthesia with 3% phenobarbital. The intravenous needle retrogradely punctures the anterior wall of the duodenum at the opening of the bile duct, inserts into the distal end of the bile duct through the duodenal papilla, both ends of the bile duct are clamped near the hepatic hilum and the bile duct opening, and 5% taurocholate (1 ml/kg) is injected into the bile-pancreatic duct. After completion, the puncture needle was pulled out after 5 min, the arterial clip and ligature were removed, and the duodenal puncture was sutured. Then the abdomen was closed and mice underwent resuscitation with normal saline (40 mL/kg) subcutaneous injection. At 48 h after the operation, the hyperlipidemia pancreatitis model was validated by amylase and triglyceride level in the serum. Afterwards, CTSS inhibitor, Z-FL-CHCHO (5 mg/kg; intraperitoneal injection; three times per day for one weeks), was used in one of these three hyperlipidemia pancreatitis groups; the other two groups were administrated PBS or none. Finally, all the mice were sacrificed and the blood and pancreatic tissues were collected for RT-PCR, western blot, and pathologic analysis.

### Construction of gene inhibition and overexpression vectors

For knockdown specific gene expression, shRNA sequences of target genes and the negative control shRNAs (scr) without sequence homology to mice genes were synthesized by GenePharma (Shanghai, China).

The CTSS expression plasmid was constructed as follows. Briefly, the restriction sites of XhoI and BamHI were inserted into the ends of the CTSS open reading frame based on primers designed from the cDNA sequences of CTSS stored in the NCBI database. The specific primers were upstream primer 5′-TAAGATGGCTGTTTTGGA TG and downstream primer 5′-TTCTTTTCCCAGATGAGACGC. Purified PCR products were ligated with a pMD18-T vector using T4 DNA ligase (Takara), and the constructed pMD18-T-CTSS plasmid was confirmed by sequencing. The pcDNA3.1 and pMD18-T-CTSS vectors were purified simultaneously with BamHI and XhoI and ligated with recombinant pcDNA3.1-CTSS to obtain the recombinant pcDNA3.1-CTSS. The DNA of pcDNA3.1-CTSS was confirmed by sequencing. Cells were transfected using Lipofectamine™ RNAiMAX Transfection Reagent (13778075, Invitrogen) following the manufacturer’s protocols.

### Enzyme-linked immunosorbent assay (ELISA)

Patients and mice plasma samples were assayed for CTSS, IL-1β, IL-10, and TNF-α levels using an ELISA kit, following the manufacturer’s instructions.

### Quantitative real-time polymerase chain reaction (qRT-PCR)

Total RNA isolation was performed using Trizol reagent according to the manufacturer’s instructions and the One-Step SYBR PrimeScript PLUS RT-RNA PCR Kit (Takara) was selected for RT-PCR analysis. The TaqMan Universal Master Mix II (Thermo Fisher Scientific, Waltham, MA, USA) was used for cDNA generation in reverse transcription. Gene relative expression was normalized to β-actin using 2^−∆∆Ct^. The experiments were performed in triplicate. Primers used in the study can be found in Table 1.

**Table 1:** Primers used in the study

Gene name	Primer sequence
CTSS	Forward:5′-TAAGATGGCTGTTTTGGATG Reverse:5′-TTCTTTTCCCAGATGAGACGC
IL-1β	Forward:5′-ATGATGGCTTATTACAGTGGCAA Reverse:5′-GTCGGAGATTCGTAGCTGGA
TNF-α	Forward:5′-GAGGCCAAGCCCTGGTATG Reverse:5′-CGGGCCGATTGATCTCAGC
IL-10	Forward:5′-AGAAGGACCAGCTGGACAACAT Reverse:5′-CAAGTAACCCTTAAAGTCCTGCAGTA
Caspase-1	Forward:5′- TTTCCGCAAGGTTCGATTTTCA Reverse:5′- GGCATCTGCGCTCTACCATC
Trypsinogen	Forward:5′-CATGAATCTACTCCTGATCC Reverse:5′- TGTCATTGTCCAGAGTCC
GSDMD	Forward:5′-CCAGCATG GAAGCCTTAGA G Reverse:5′-CAGAGTCGAGCACCAGACAC
CD11c	Forward:5′-TTC TTC TGC TGT TGG GGT TTG Reverse:5′-CAA CCA CCA CCC AGG AAC TAT
CD206	Forward:5′-CTACAAGGGATCGGGTTTATGGA Reverse:5′-TTGGCATTGCCTAGTAGCGTA
IRF1	Forward:5′-CCCAAGCTTATGCCCATCACTCGGATGC Reverse:5′-CCGCTCGAGCTACGGTGCACAGGGAATGG
IRF3	Forward:5′-ACCGACGGCTTTACAGAGAA Reverse:5′-TCCTCGTCACTGCAGTCTTT
IRF4	Forward:5′-CCATGACAACGCCTTACCCT Reverse:5′-TTATGCTTGGCTCTGTGGGG
IRF5	Forward:5′-GTTGCCTTTGACGGACCTA Reverse:5′-GTTGCCTTTGACGGACCTA
IRF7	Forward:5′-ATATCTCACGTGACCGAGGA Reverse:5′-AGCTGATGGTGCTGGAAGTC
IRF8	Forward:5′-CGAGGTTACGCTGTGCTTTG Reverse:5′-TTATGCTTGGCTCTGTGGGG
β-actin	Forward:5′- GAGACCTTCAACACCCC; Reverse:5′- GTGGTGGTGAAGCTGTAGCC

### Western blot analysis

Western blot analysis was performed using 15% sodium dodecyl sulfate–polyacrylamide gel electrophoresis (SDS-PAGE) and transferred onto a nitrocellulose membrane. The blots were incubated with primary antibodies overnight at 4 °C. Following three washes, membranes were then incubated with secondary antibody overnight at 4 °C. Signals were visualized with ECL and exposed using a ChemiDoc XRS imaging system (BioRad, Hercules, CA, USA). In order to save the amount of antibody, we kept the gels that were labeled with the loaded protein before incubating the antibody. The blots in our figures were cut and cropped gels in the images retain important bands.

### Flow cytometry analysis

Flow cytometry was used for the detection of polarization in macrophage phenotypes. Macrophage stimulated by PA (500 μM) for 24 h, were washed with pre-cooling buffer (1% bovine serum albumin in PBS containing 0.01% NaN3, Thermo Fisher Scientific) and incubated with 10% mouse serum for 20 min. Then, the cells were incubated with the LIVE/DEAD Fixable Dead Cell Stain Kit (Thermo Fisher Scientific), CD206-APC (Invitrogen), and CD11C-PE (BD) at the manufacturer’s recommended dilution for 40 min at 4 °C. The samples were then washed, centrifuged at 1,500 × g for 5 min and resuspended in PBS, and then analyzed with a FACS Canto II system and BD FACS DIVA software.

### Dual-luciferase activity assay

PCR was performed to obtain the CTSS UTR target site. A luciferase reporter with CTSS UTR and a putative IRF5 binding site was established in a pMiRreport vector. Macrophage were co-transfected with a recombinant and mutant recombinant CTSS promoter with the IRF5 binding site using Lipofectamine 3000. Luciferase reporter assays were performed using a dual-luciferase assay system (Promega Madison, WI, USA).

### Electrophoretic mobility shift assay (EMSA)

EMSA was used to verify the interaction between IRF5 and the CTSS gene. The specific promoter fragments of CTSS containing the GCAAAC-box and mutated GCAAAC-box were synthesized as biotin end-labeled and unlabeled oligonucleotides. The unlabeled GCAAAC-box oligonucleotides served as a competitor. The anti-IRF5 antibody was used for supershift identification. The assay was performed using the LightShift Chemiluminescent EMSA Kit (Thermo Fisher Scientific) according to the manufacturer’s instructions.

### Isolation and identification of exosomes

ExoQuick-TC (SBI Biotech, Tokyo, Japan) and total exosomal isolation were performed according to the manufacturer's instructions. Briefly, the collected cell culture medium was centrifuged at 2000 × *g* for 30 min and the supernatant was collected. One-fifth of the ExoQuick-TC exosomal precipitate or half of the total exosomal isolate was added to the supernatant and the suspension was incubated overnight at 4 °C. The suspensions were centrifuged at 1,500 × *g* for 30 min for ExoQuick-TC or at 10,000 × *g* for 60 min for total exo-some isolation. They were re-suspended in PBS. Before further experiments, exosomal marker proteins were identified. The exosomes were re-suspended in Trizol for RNA analyses; or in lysis buffer (8 M urea/2.5% SDS, 5 μg/mL leupeptin, 1 μg/mL pepstatin, and 1 mM phenylmethylsulphonyl fluoride) for protein analyses or used for transmission electron microscopy.

### Scanning electron microscopy

Fixed specimens were placed on a 400-mesh carbon/Formvar coated grid at optimal concentration and allowed to absorb into the formvar for 1 min. For immunogold staining, the raster was placed in blocking buffer for 1 h in the blocking/permeation step. Without rinsing, the raster was immediately added to the primary antibody dilution (1:300 anti-CD9 ab92726, Abcam and anti-GPC1 PIPA528055, Thermo Fisher Scientific) overnight at 4 °C. As a control, some grids were not exposed to the primary antibody. The next day, the grids were washed with PBS and then floated in 10 nm gold particles (Aurion, Hatfield, PA, USA) ligated with appropriate secondary antibodies and incubated at room temperature for 2 h. They were then rinsed with PBS and placed in 2.5% glutaraldehyde in 0.1 M phosphate buffer for 15 min. The grids were rinsed in PBS and distilled water, dried, and stained with uranyl acetate. Samples were photographed with a Tecnai Bio Twin transmission electron microscope (FEI, Hillsboro, OR, USA) and images were taken with an AMT CCD camera (Advanced Microscopy Technology, Danvers, MA, USA).

### Hematoxylin and eosin (HE) staining

The pancreas was dissected along the pancreatic duct and a 0.5 cm × 0.5 cm specimen was fixed in 4% paraformaldehyde solution. After embedding in paraffin, the samples were cut into 4-μm-thick sections and stained with HE. Then, the sections were observed under an optical microscope and photographed.

### Immunohistochemical assay

Pancreatic acinar cells were cultured in 24-well plates. Cells were fixed with 4% paraformaldehyde for 10 min at room temperature and then washed three times in PBS containing 0.1% Tween-20 to permeabilize cells. Cells were then blocked with 5% BSA for 30 min in PBS. Primary antibodies were incubated at 1:100 in PBS with 1% BSA and 0.05% Tween-20 at 4 °C overnight. Secondary antibodies were incubated at room temperature for 2 h. Images were taken with a Zeiss Axiovert 40 CFL.

### TUNEL assay

Terminal deoxynucleotidyl transferase dUTP nick end labeling (TUNEL) was performed to stain the mice pancreas sections (Roche, Shanghai, China). Pancreatic acinar cells positive for TUNEL were counted in 20 fields with × 200 magnification.

### MTT assay

The viability of pancreatic acinar cells was tested using CCK8 assay according to the manufacturer’s instructions. After transfection and treatment, cells were incubated in six-well plates and 10 μL cell counting kit-8 solutions were added to each well. Cells were incubated for 2 h at 37 °C, and absorbance was measured at 450 nm with a spectrophotometer.

### Immunofluorescence assay

To conduct immunohistochemical analyses, samples were blocked in normal goat serum with 5% BSA in TBS for 1 h at room temperature. The sections were incubated with primary antibody at a dilution of 1:400 overnight at 4 °C and then washed with PBS three times. After incubation with secondary antibodies (16 h), the sections were subjected to a DAB reaction. The sections were photographed using a digitalized microscope camera (Nikon, Tokyo, Japan).

### Transcription site prediction

Sequences were submitted to the JASPAR database (http://jaspar.genereg.net/) for transcription site prediction.

### Statistical analysis

The data are presented as the means ± standard error of the mean (SEM). Tukey’s test or Student’s t-test for unpaired results was used to evaluate the differences among more than three groups or between two groups, respectively. Differences were considered significant for values of *P* < 0.05.

## Results

### Free fatty acid-mediated macrophage M1 polarization in vitro

**Fig. 1:**
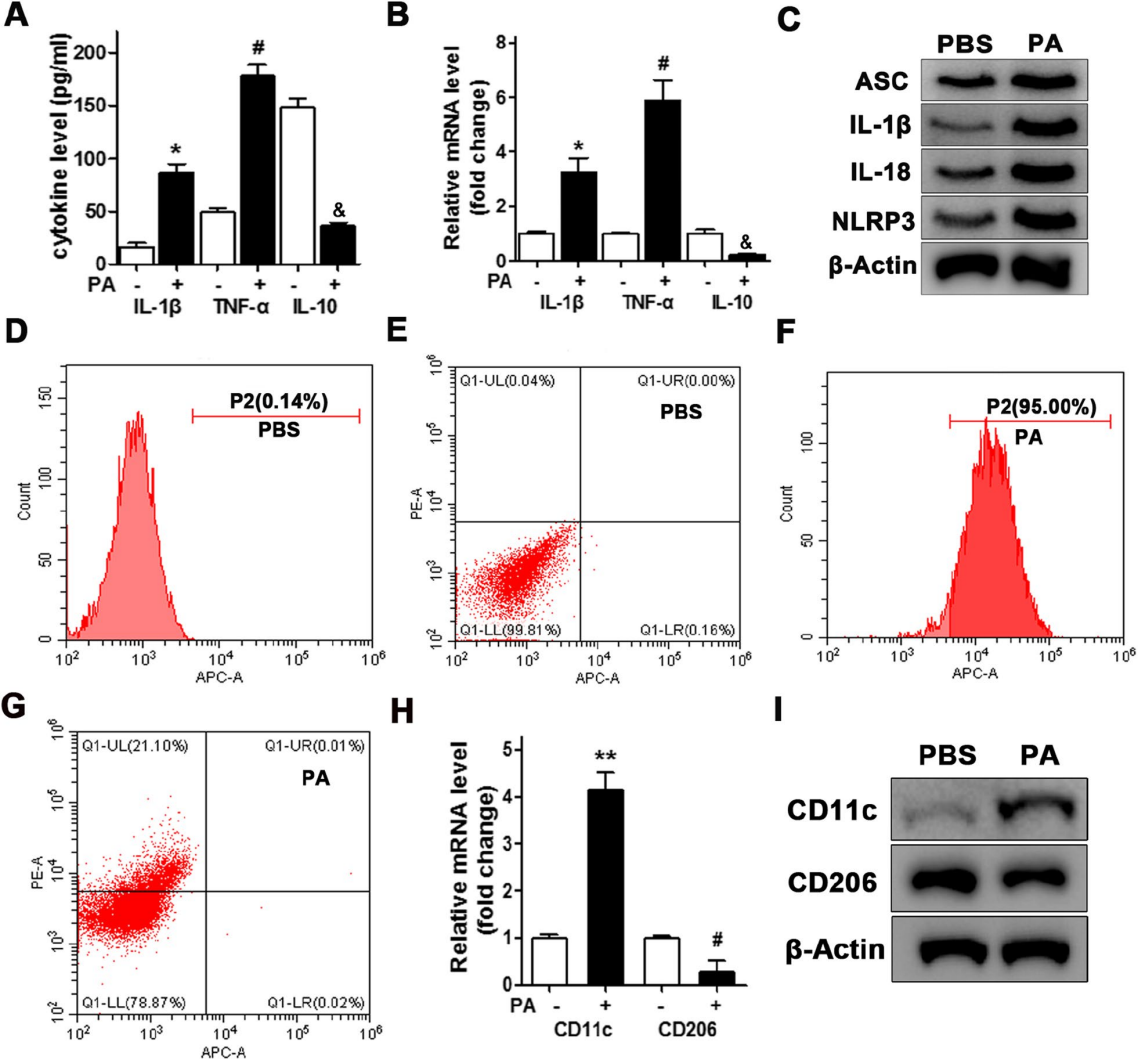
Free fatty acid-mediated macrophage M1 polarization in vitro. Mouse abdominal macrophage were isolated by high-sugar DMEM and then inoculated into a sterile Petri dish (2.5 × 10^5^/ml). Palmitic acid (PA, 500 μM) was used for fatty acid stimulation. **A** Inflammation factors were detected by ELISA in macrophage culture medium, which was stimulated by PA, and **B** quantitative cellular expression was measured. Data are shown as the mean ± SEM, n = 3. *P < 0.05, ANOVA. Protein levels of ASC, IL-1β, IL-18 and NLRP3 were performed by western blot in macrophage stimulated by PA (**C**). Flow cytometry to assess polarization in macrophage stimulated by PBS (**D**, **E**) or PA (**F**, **G**). Anti-F4/80 (**D**, **F**), anti-CD11c, and anti-CD206 (**E**, **G**). CD11c indicates M1 macrophage, CD206 indicates M2 macrophage. Marker genes of macrophage polarization were detected by qRT-PCR (**H**) and western blotting (**I**). Data are shown as the mean ± SEM, n = 3. *P < 0.05, ANOVA

We used mouse abdominal macrophage stimulated by PA to determine the effects of FFA on the expression levels of cytokines in vitro. In macrophage stimulated by PA, the levels and expression of the pro-inflammatory cytokines IL-1β and TNF-α increased significantly, whereas the level of the anti-inflammatory cytokine IL-10 decreased (Fig. 1A, B). Protein levels of ASC, IL-1β, IL-18 and NLRP3 are all increased in macrophage stimulated by PA (Fig. 1C). We then used flow cytometry to analyze the effects of FFA on macrophage polarization in vitro (Fig. 1D–G). M1 or M2 positive cells were detected using the markers F4/80, CD11c, and CD206. F4/80+, CD11c + , CD206 − indicated M1 positive cells whereas F4/80+, CD11c−, CD206+ indicated M2 positive cells. The level of CD11c was increased in macrophage stimulated by PA, whereas levels of CD206 were decreased (Fig. 1H, I). Therefore, the increase in the ratio of CD11c relative to CD206 indicates that FFA mediates M1 polarization in macrophage.

### Transcription factor IRF5 regulates CTSS expression in pro-inflammatory macrophage

**Fig. 2:**
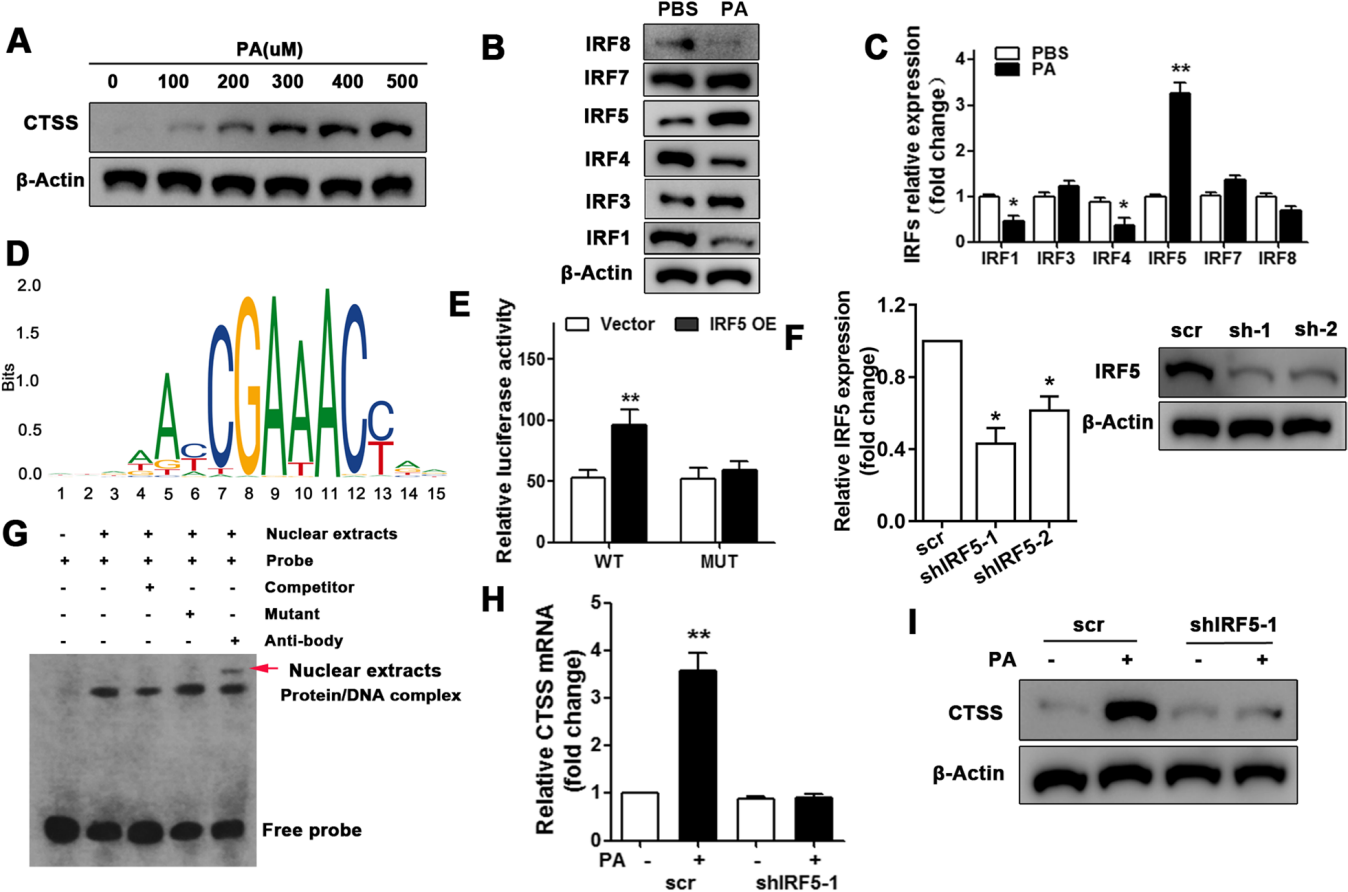
Transcription factor IRF5 is responsible for CTSS expression in proinflammatory macrophage*.* Western blot analysis (**A**) for CTSS expression in macrophage with increased concentrations of palmitic acid (PA, 0, 100, 200, 300, 400, 500 μM). Western blot (**B**) and qRT-PCR (**C**) analysis for the expression of IRF family gene expression under 500 μM PA. JASPAR (http://jaspar.genereg.net/) prediction of the IRF5 transcription binding site in the CTSS promoter (**D**). Dual-luciferase reporter system was performed with WT and MUT CTSS promoter with IRF5 overexpression (**E**). qRT-PCR and western blot analysis (**F**) for the efficiency of IRF5 gene knockdown in macrophage. An electrophoretic mobility shift assay (EMSA) was used to assess the IRF5 and CTSS interaction (**G**). qRT-PCR (**H**) and western blot analysis (**I**) for CTSS expression when transfected with scr or shIRF5-1. Data are shown as the mean ± SEM, n = 3, ANOVA. *P < 0.05, **P < 0.05

To further characterize CTSS in HP the level of its expression was determined in macrophage stimulated by PA. The expression of CTSS increased when the concentration of PA increased in macrophage culture medium (Fig. 2A), confirming that it may be associated with HP. The expression levels of several IRF family proteins were also determined, these included IRF1, 3–5, 7, and 8. According to western blot and qRT-PCR analysis, the expression of IRF5 was significantly higher in response to PA whereas the expression of IRF1 and IRF4 was significantly lower (Fig. 2B, C). No significant difference was found in the expression of IRF3, 7, and 8. JASPAR was used to predict the transcription binding site of IRF5 in the CTSS promoter and a dual-luciferase reporter was used to confirm the predicted site in wild-type and mutated CTSS promoter in the presence of IRF5 overexpression vector (Fig. 2D, E). EMSA was used to visualize the interaction between IRF5 and CTSS (Fig. 2F). RNA interference was used to down-regulate IRF5 in macrophage stimulated with PA (Fig. 2G, H). CTSS expression was significantly lower in macrophage stimulated with PA when the expression of IRF5 was down-regulated (Fig. 2I, J). These results provide evidence that IRF5 up-regulate CTSS in M1 macrophage in response to FFA stimulation.

## Exosome-derived CTSS induces injury in acinar cells via trypsinogen and caspase-1

**Fig. 3:**
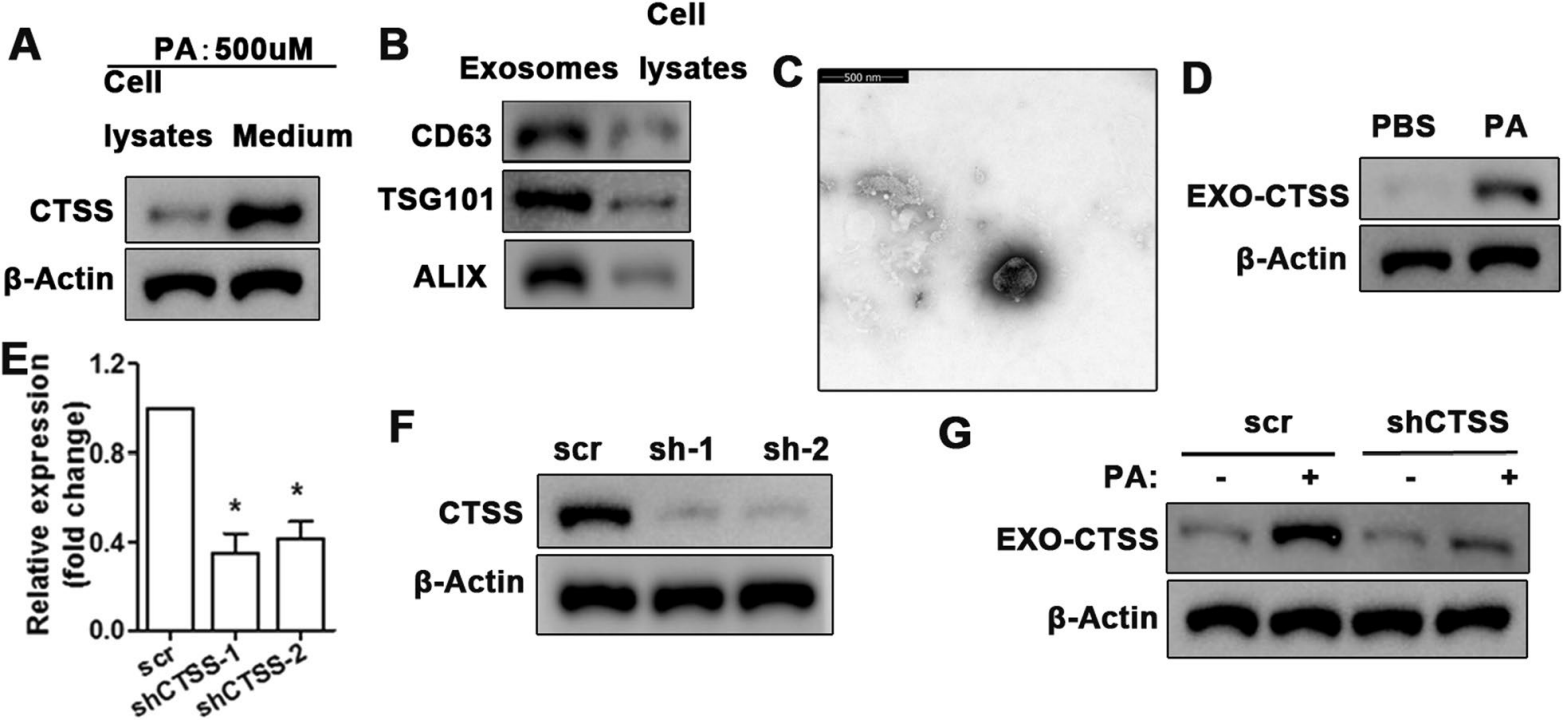
CTSS is produced in macrophage and transported by exosomes. Western blot analysis of CTSS in macrophage cell lysates and medium after PA (500 μM) stimulation for 24 h (**A**). Western blot analysis of exosome-specific proteins in exosomes and cell lysates detected 24 h after palmitic acid (PA, 500 μM) stimulation of macrophage (**B**). Electron microscopy of exosomes (**C**). (Scale bars = 500 nm) Exosome-derived CTSS from PA (500 μM) stimulated macrophage (**D**). QRT-PCR (**E**) and western blot (**F**) analysis of CTSS gene knockdown efficiency in macrophage. Data are shown as the mean ± SEM, n = 3, ANOVA. *P < 0.05. Detection of CTSS in exosomes from PA (500 μM) stimulated macrophage; macrophage were transfected with shCTSS, scr transfection as the control (**G**)

Having established that CTSS up-regulated by IRF5 in macrophage in response to PA, we tried to determine whether CTSS is transported in exo-some. CTSS was found at a higher level in the medium than in cell lysates after stimulation by PA (Fig. 3A). After 24 h stimulation with PA, exosomes were isolated from macrophage culture medium and exosome-specific proteins (CD63, TSG101, and ALIX) were detected by western blotting (Fig. 3B). Results indicate that the level of exosome-maker proteins were higher in samples isolated from medium than from cells lysates. To support these results, the presence of exosomes was confirmed by electron microscopy (Fig. 3C). High levels of CTSS were found in exosomes produced from PA-stimulated macrophage (Fig. 3D). The interference of CTSS mRNA in macrophage stimulated with PA reduced the level of CTSS in exosomes (Fig. 3E–G).

**Fig. 4:**
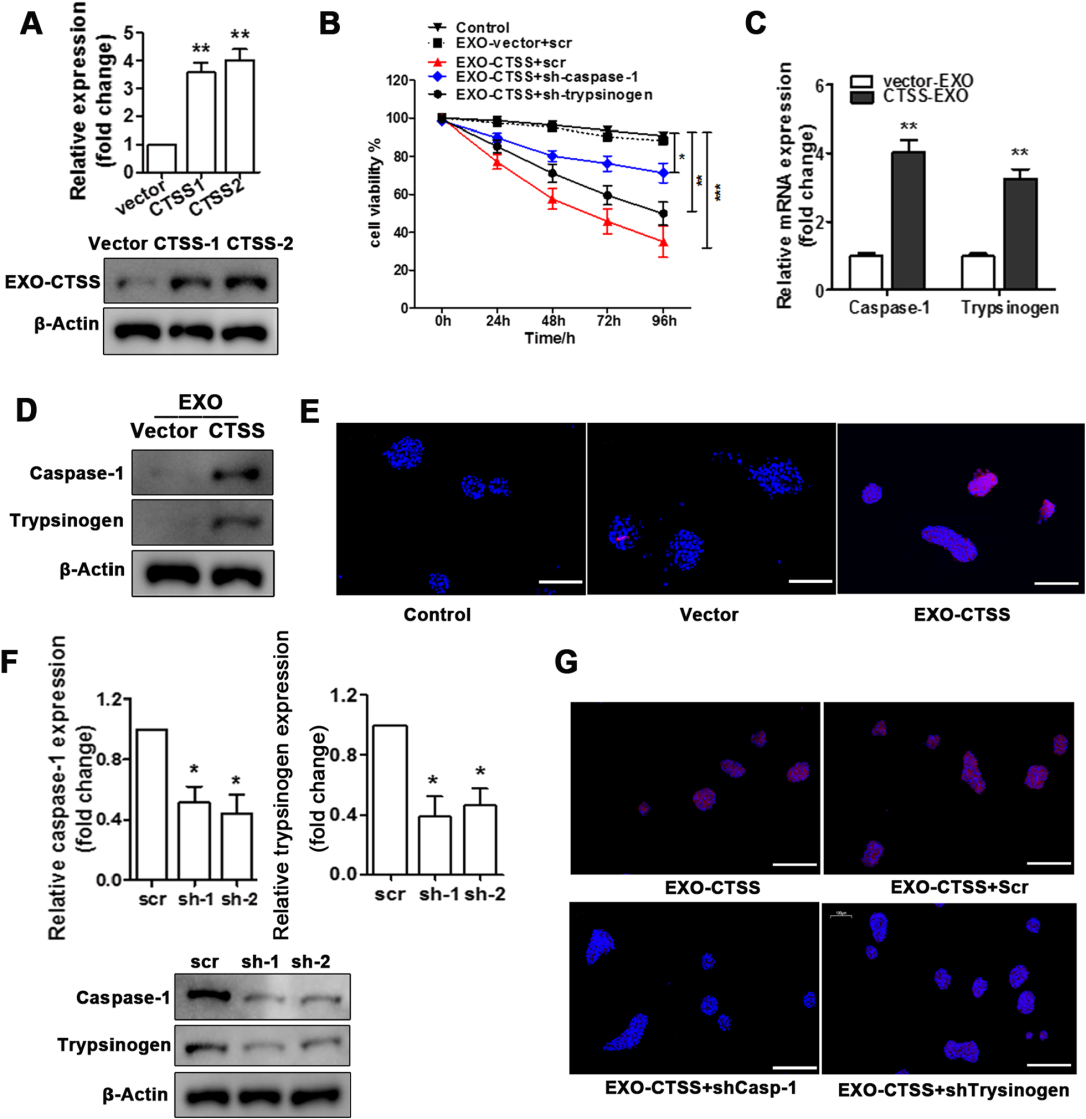
Macrophage exosome-derived CTSS induced acinar cell injury via trypsinogen and caspase-1. QRT-PCR and western blot analysis (**A**) of CTSS overexpression in macrophage, empty vector are used as the control. Pancreatic acinar cell viability over time in control, EXO-vector + scr, EXO-CTSS + scr, EXO-CTSS + shCaspase1, and EXO-CTSS + shtrypsinogen (**B**). QRT-PCR (**C**) and western blot analysis (**D**) for caspase-1 and trypsinogen expression of acinar cells after EXO-CTSS stimulation. TUNEL assay for acinar cells apoptotic after EXO-CTSS treatment, with empty vector control, and normal blank control. (Scale bars = 100 μm) (**E**). QRT-PCR and western blot analysis (**F**) of the efficiency of caspase-1 and trypsinogen gene knockdown in acinar cells, with scr control. TUNEL assay to assess apoptosis after EXO-CTSS treatment in acinar cells, which are transfected with scr, shCaspase1, or shTrypsinogen, no transfection is the blank control. (Scale bars = 100 μm) (**G**). Data are shown as the mean ± SEM, n = 3, ANOVA. *P < 0.05, **P < 0.01, ***P < 0.001

To further investigate the effect of CTSS in acinar cells, CTSS overexpression system was established in macrophage (Fig. 4A).The presence of CTSS in exosomes reduced the viability of pancreatic acinar cells (Fig. 4B). Additionally, the relative RNA expression and protein levels of caspase-1 and trypsinogen were significantly increased in acinar cells after treatment with exosomes that contain CTSS (EXO-CTSS) (Fig. 4C, D). TUNEL assay indicates that the viability of acinar cells was decreased when they were co-culture with EXO-CTSS (Fig. 4E). Next, the RNA interference of trypsinogen and caspase-1 was performed in acinar cells (Fig. 4F). Interestingly, the downregulation of either caspase-1 or trypsinogen rescued cell viability in pancreatic acinar cells treated with EXO-CTSS, but the viability did not reach the same level as in cells treated with control exosomes without CTSS overexpressing (Fig. 4C). Furthermore, the survival of acinar cells also improved when caspase-1 or trypsinogen were down-regulated (Fig. 4G). These results demonstrate that CTSS delivered by exosome can up-regulates caspase-1 and trypsinogen in acinar cells to promote cell death.

### Macrophage exosome-derived CTSS induced pyroptosis in vitro and in vivo

**Fig. 5:**
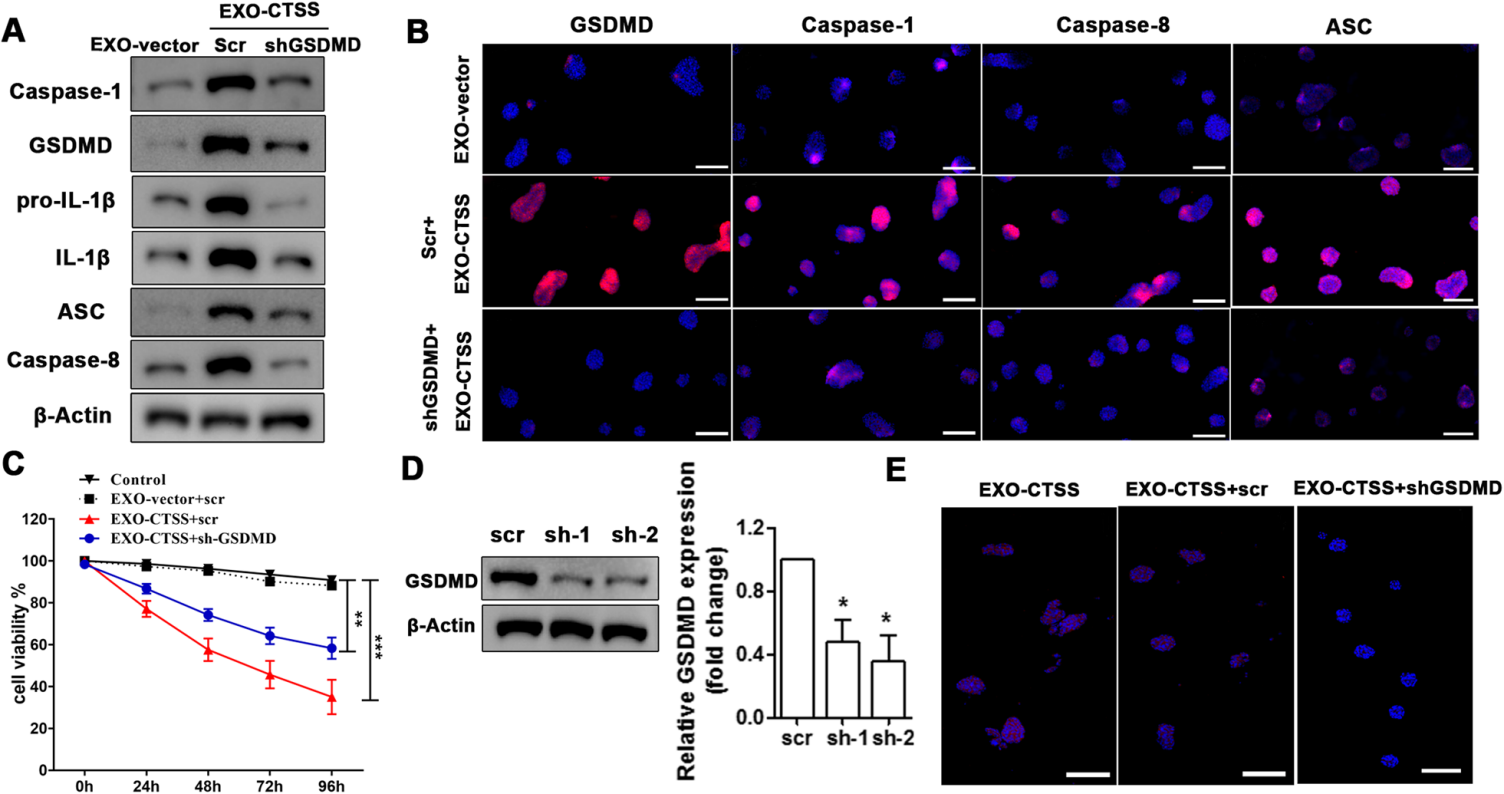
Macrophage exosome-derived CTSS induced pyroptosis in vitro. Western blot analysis of caspase-1, GSDMD, pro-IL-1β, IL-1β, ASC, and caspase-8 in acinar cells, which are treated with EXO-CTSS or EXO-vector and transfected with scr or shGSDMD (**A**). Immunofluorescence staining for GSDMD, caspase-1, caspase-8, and ASC in acinar cells are treated with EXO-CTSS or EXO-vector, and transfected with scr or shGSDMD (**B**). The location and level of GSDMD, caspase-1, caspase-8, and ASC are shown by red fluorescence, and nucleiare stained with DAPI. QRT-PCR and western blot analysis (**C**) for the efficiency of GSDMD gene knockdown in acinar cells, scr as the control. Acinar cells viability over time in control, EXO-vector + scr, EXO-CTSS + scr, and EXO-CTSS + shGSDMD (**D**). TUNEL assay for apoptosis was performed in acinar cells after EXO-CTSS treatment; cells were transfected with scr or shGSDMD. (Scale bars = 100 μm). (**E**). Data are shown as the mean ± SEM, n = 3, ANOVA. *P < 0.05, **P < 0.01, ***P < 0.001

To determine whether pyroptosis was activated by CTSS, we first characterized the expression of GSDMD (key pyroptosis protein), Caspase-1, Caspase-8 (pyroptosis switch, ASC (effector protein of pyroptosis), and pro-inflammatory proteins pro-IL-1β and IL-1β in acinar cells treated with CTSS enveloped by exosomes (Fig. 5A). Western blot analysis showed that CTSS enhanced the level of pro-inflammatory proteins (pro-IL-1β, IL-1β) and pyroptosis pathway proteins (GSDMD, Caspase-1, Caspase-8, and ASC). Furthermore, the expression of pyroptosis pathway proteins was indicated by immunofluorescence (Fig. 5B). The fluorescence intensity was increased in acinar cells with EXO-CTSS processing. We also determined acinar cell viability with and without GSDMD and found that cell viability is significantly lower in cells treated with exogenous CTSS compared with untreated cells. However, cell viability improved when GSDMD down-regulate (Fig. 5C). Next, the pyroptosis pathway was blocked by the RNA interference of GSDMD (Fig. 5D). The levels of pyroptosis pathway proteins were significantly reduced in acinar cells when GSDMD was silenced even with EXO-CTSS treatment (Fig. 5A, B). TUNEL assays confirmed that downregulating GSDMD in cells treated with exosome-derived CTSS decreases the level of apoptosis that occurs in acinar cells expressing GSDMD (Fig. 5E). Overall these results demonstrate that CTSS derived from macrophage exosome induces pyroptosis activation in vitro.

### Free fatty acids and pancreatic inflammation in HP mouse model

**Fig. 6:**
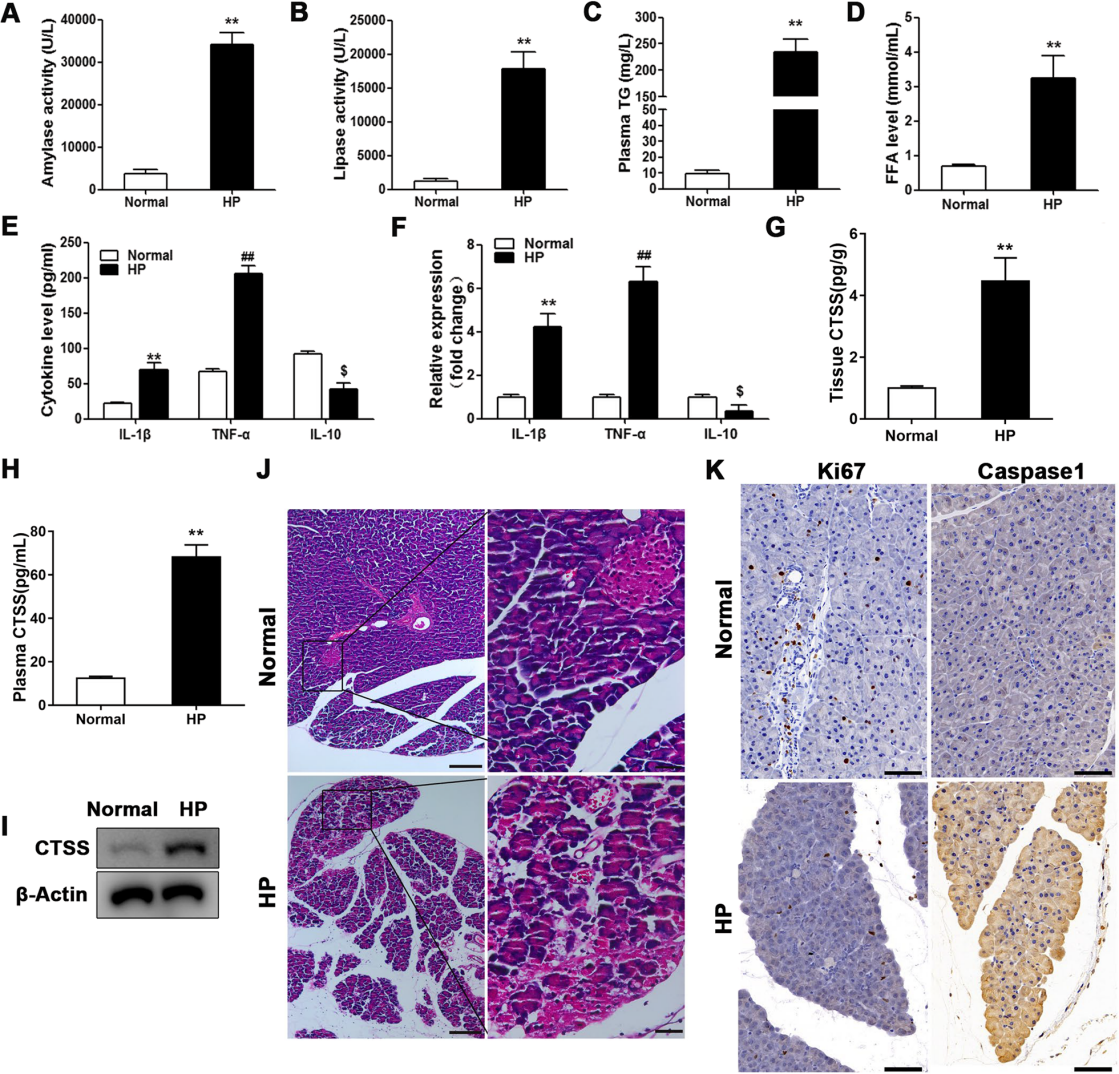
Increased free fatty acids and pancreatic inflammation in a mouse model of hyperlipidemic pancreatitis (HP). A mouse model of HP was constructed in C57BL/6 J mouse (n = 6); and control (n = 6). After 6 weeks, mice were euthanized, and peripheral blood and pancreas tissue were collected. **A** Amylase and **B** lipase activity, **C** Triglyceride and **D** free fatty acid (FFA) levels in peripheral blood were measured by ELISA. **E** Plasma inflammation factors (IL-1β, TNF-α, and IL-10) detection by ELISA and **F** qRT-PCR. Data are shown as the mean ± SEM, n = 6. *P < 0.05, **P < 0.01, ANOVA. CTSS level in HP mouse peripheral blood (**G**) and pancreas tissue (**H**). Data are shown as the mean ± SEM, n = 6, ANOVA. **P < 0.01. Western blot (**I**) of CTSS in HP mouse pancreas tissues. Pathologic analyses were performed in pancreas tissue samples. **J** Hematoxylin–eosin (HE) staining shows the tissue injury of HP mouse (Scale bars = 200 μm (left); Scale bars = 50 μm (right)), **K** present the expression of Ki67 and caspase-1 (Scale bars = 50 μm)

To investigate the characteristics of pancreatic inflammation in vivo, we constructed an HP mouse model in C57BL/6 J mice by drug administration. The amylase, lipase, TG and FFA levels in the peripheral blood and pancreas tissue were found to be elevated in HP mice compared to the normal control (Fig. 6A–D). The levels and expression of the pro-inflammatory cytokines IL-1β and TNF-α were also elevated in the HP mouse model whereas levels of the anti-inflammatory cytokine IL-10 were decreased (Fig. 6E, F). We also assessed levels of CTSS in the pancreatic tissue and peripheral blood in the mouse model of HP (Fig. 6G–I). CTSS expression and protein levels of CTSS were found to increase significantly in both the peripheral blood and tissue of the HP model. The damage in pancreatic tissue was evaluated in the HP mouse model. HE staining indicated the degradation of acinar cells, shrinkage of islets of Langerhans, and increased intralobular spaces in the pancreatic tissue of HP mice compared with that of normal mice (Fig. 6J). Moreover, levels of the cell division marker Ki67 were higher in normal pancreas, which indicates that lower cell proliferation occur in HP pancreatic tissue (Fig. 6K). Immunohistochemistry demonstrates a higher activity of caspase-1 in HP mouse tissue compared with normal tissue (Fig. 6K), which signifies the possible activation of pyroptosis-related proteins in the pancreas of HP mice. Overall, these results demonstrate that the inflammatory characteristics in pancreas of HP mice.

### Macrophage exosome-derived CTSS induced pyroptosis in vivo

**Fig. 7:**
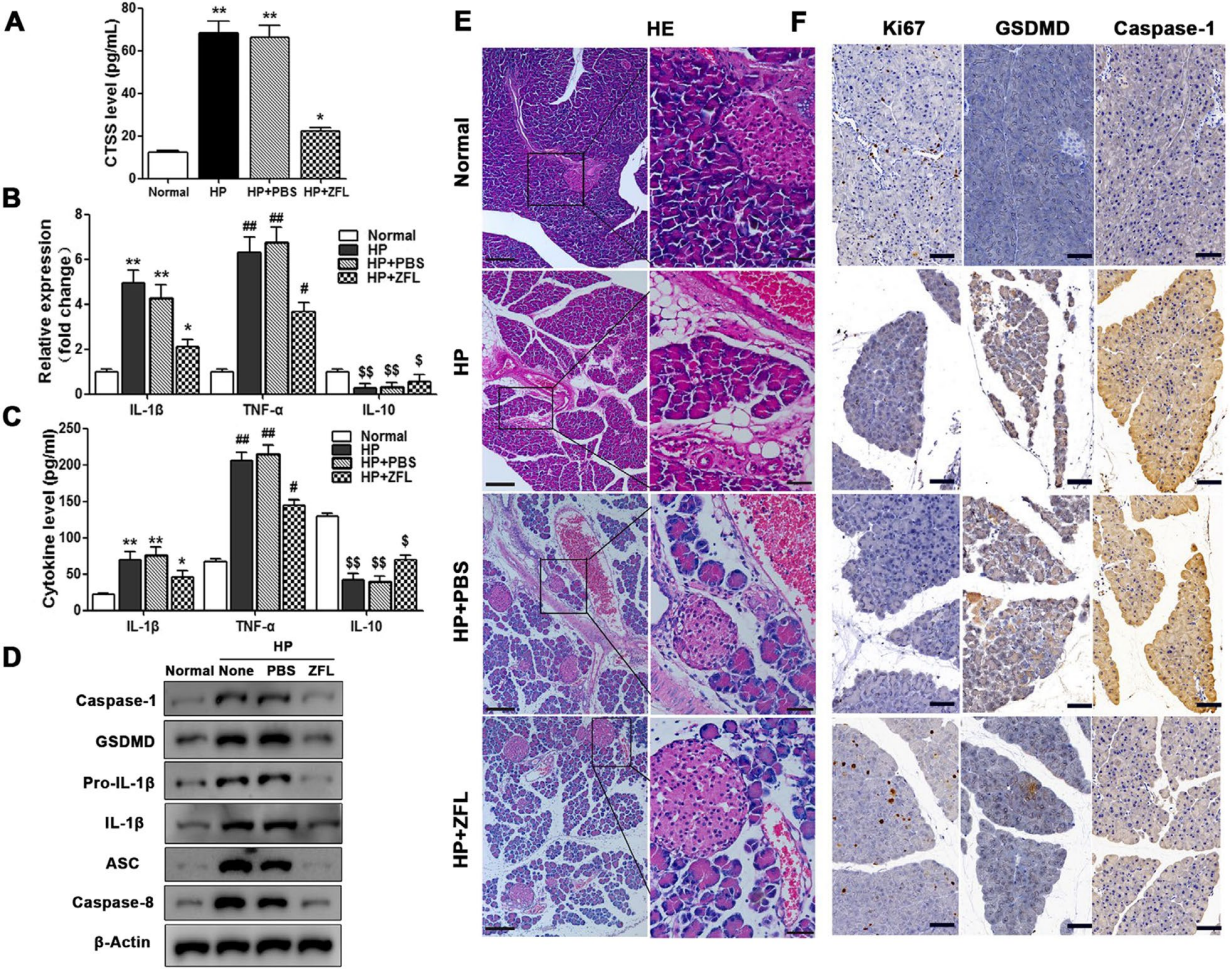
Macrophage exosome-derived CTSS induced pyroptosis in vivo. HP mice were divided into different groups, normal, HP, HP + PBS, and HP + Z-FL-COCHO. ELISA (**A**, **B**) QRT-PCR detection (**C**) for CTSS and inflammation factor levels in the peripheral blood of the HP mouse model. Proteins level of caspase-1, GSDMD, pro-IL-1β, IL-1β, ASC, and caspase-8 were assessed by western blotting, in HP mice with different treatments (**D**). Pathologic analysis for HP mice with different treatments, including HE (**E**) shows the pathology (Scale bars = 200 μm (left); Scale bar = 50 μm (right), and Ki67, immunohistochemistry of GSDMD and caspase-1. Scale bar = 50 μm (**F**). Data are shown as the mean ± SEM, n = 6, ANOVA. *P < 0.05, **P < 0.01, ***P < 0.001

We assess the role of CTSS in HP mouse model, CTSS inhibitor, Z-FL-COCHO, was used for CTSS knockdown. The mice were divided into different groups: normal mice, HP mice, and HP mice with or without Z-FL-COCHO administration. After 2 weeks treatment, the levels of CTSS and cytokines, and relative expression of cytokines were measured in peripheral blood. The data shows that CTSS levels are clearly increased in HP mouse, but are lowered with Z-FL-COCHO is present (Fig. 7A). The levels of IL-1β and TNF-α were significantly elevated in response to HP whereas the levels of IL-10 were reduced. CTSS inhibitor reduced the levels of IL-1β and TNF-α but they were still significantly higher than in the control (Fig. 7B, C). Protein levels of caspase-1, GSDMD, pro-IL-1β, IL-1β, ASC, and caspase-8 were all increased in the HP mice compared with the control but lower levels were found when a CTSS inhibitor is used (Fig. 7D). Pathologic analysis of pancreatic tissue from the HP mouse model with CTSS inhibition demonstrated that CTSS aggravates the level of injured pancreatic tissue (Fig. 7E), reduces cell proliferation, and increased the expression of GSDMD and caspase-1 (Fig. 7F). These results confirm the findings in vitro, that CTSS is involved in the induction of pyroptosis in the pancreas of the HP mouse model in vivo.

### CTSS up-regulated in patients with hyperlipidemia pancreatitis

**Fig. 8:**
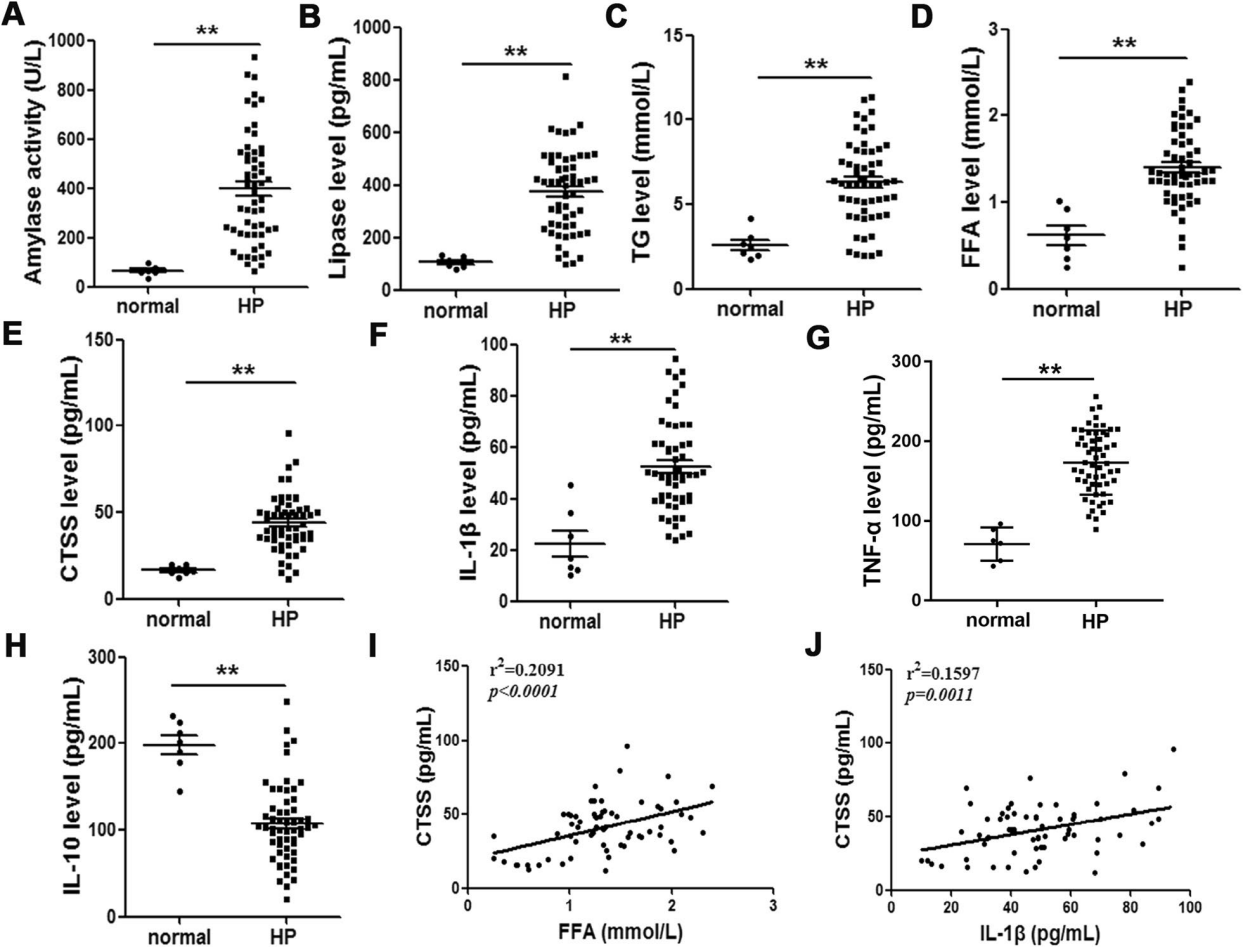
CTSS is up-regulated in peripheral blood of hyperlipidemic pancreatitis (HP) patients and in an HP mouse model. Amylase level (**A**), lipase level (**B**), TG level (**C**), FFA level (**D**), CTSS level (**E**), IL-1β (**F**), TNF-α (G) and IL-10 (**H**) levels in the peripheral blood of HP patients. Data are shown as the mean ± SEM, HP (N_HP_ = 57), control (n_n_ = 7), ANOVA. **P < 0.01. Linear correlation analysis of CTSS and FFA (**I**), CTSS and IL-1β (**J**)

The peripheral blood of patients with hyperlipidemia pancreatitis were sampled, and the amylase, lipase and TG level were measured by ELISA kit. The results show that amylase, lipase and TG were both elevated in HP patient’s serum (Fig. 8A–C). To determine the extent of CTSS involvement in HP we measured its levels in these patients. FFA, CTSS, IL-1β, TNF-α and IL-10 increased significantly whereas level of IL-10 decreased in HP (Fig. 8D–H). Furthermore, a positive correlation was found between CTSS and FFA, and between CTSS and IL-1β (Fig. 8I, J). These results suggest that CTSS may have an important role in the progression of inflammasome relevant HP.

## Discussion

Cathepsins are responsible for regulating the premature release of trypsinogen that initiates the onset of pancreatitis [18, 25]. CTSB, CTSL, and CTSS are known to be up-regulated in pancreatitis, and although the roles of CTSB and CTSL are well-established that of CTSS is less known [25]. In this study we examined the contribution of FFA induced macrophage-derived CTSS on pyroptosis in pancreatic cells.

We found that the levels of CTSS, FFA, and IL-1β increase with the severity of HP whereas levels of IL-10 decreased and a positive correlation existed between CTSS and FFA and between CTSS and IL-1β. These results demonstrate that CTSS was up-regulated by the levels of FFA and that macrophage adopt an M1 pro-inflammatory phenotype [11]. Studies that report an association between CTSS and FFAs are rare in the literature. However, the effect of PA on the inflammatory response in macrophage is well-documented. PA is known to increase levels of IL-1β mRNA by activating NF-κB [30]. Moreover, the overexpression of IL-1β induces chronic pancreatitis in mice [31]. In contrast, the anti-inflammatory IL-10 is down-regulated. IL-10 is known to have a positive effect on pancreatitis by inhibiting the macrophage release of inflammatory mediators and preventing the development of acinar necrosis [32, 33].

We also examined whether any of the IRF family of transcription factors promote the expression of CTSS in response to PA. We found that IRF5 was expressed significantly higher in response to PA whereas the expression of IRF1 and IRF4 was significantly lower. This corresponds with studies associated with macrophage polarization [34]. IRF5 participates in the promotion of pro-inflammatory M1 macrophage whereas IRF-3 and IRF-4 promote M2 polarization. Therefore, PA induces the production of M1 macrophage. In addition, through a dual-luciferase and EMSA assay, we found that IRF5 binds to the promoter of CTSS and up-regulates CTSS expression in response to PA. The expression of CTSS was lower in macrophage stimulated with PA when IRF5 was inhibited. Therefore, our results demonstrate that IRF5 up-regulates CTSS in M1 macrophage in response to FFA.

We next determined whether CTSS was transported by exosome and if exosomes overexpressing CTSS could influence the viability of acinar cells, which would indicate whether CTSS may be involved in pancreatic injury. The overexpression of CTSS in exosomes reduced the viability of pancreatic acinar cells and the levels of caspase-1 and trypsinogen were significantly increased. The up-regulation of caspase-1 and trypsinogen in acinar cells demonstrates that CTSS overexpression could promote pyroptosis. The overexpression of caspase-1 and trypsinogen has been linked previously to inflammatory pancreatic diseases [35]. Caspase-1 forms part of the NLRP3 inflammasome with ASC. In response to FFA, the inflammasome proteolytically activates pro-IL-1β and pro-IL-18 to generate the active forms of IL-1β and IL-18 [36]. Decreasing the level of FFA with a lipase inhibitor can reduce levels of IL-1β and ameliorate the extent of pancreatic damage [37]. Trypsinogen is involved in the initiation of AP [38]. The up-regulation of trypsinogen by CTSS indicates that CTSS may be involved in the preliminary stages of HP.

Finally, we determined whether CTSS was involved in pancreatic acinar cell pyroptosis by measuring the expression of caspase-1, caspase-8, ASC, and pro-inflammatory proteins pro-IL-1β and IL-1β in acinar cells incubated with exosome expressing CTSS and in an HP mouse model with CTSS inhibition. GSDMD expression triggers pyroptosis through the regulation of IL-1β and IL-18 [39]. In this study, we found that the levels of pro-inflammatory proteins where enhanced in acinar cell treated by exosome-derived CTSS but silencing GSDMD significantly reduced the levels. Likewise, cell viability is significantly lower in cells treated with exogenous CTSS but improves when GSDMD down-regulated. In the murine HP model, the relative expression and levels of IL-1β and TNF-α were significantly elevated in peripheral blood whereas the levels of IL-10 were reduced. The CTSS inhibitor reduced the levels of IL-1β and TNF-α whereas levels of IL-10 increased. Protein levels of Caspase1, GSDMD, pro-IL-1β, IL-1β, and ASC are all increased in HP mice but lower levels are found when a CTSS inhibitor is used. Pathologic analysis of pancreatic tissue from the HP mouse model with or without CTSS inhibition demonstrated that CTSS increases the level of injured pancreatic tissue, reduces cell proliferation and increases the level of apoptosis and the expression of Caspase-1. Caspase-1 is activated by the inflammasome complex and initiates pyroptosis by cleaving GSDMD [40]. The loss of CTSS inhibits the promotion of the inflammasome whereas the loss of GSDMD, which is a substrate of caspase-1, prevents cells from entering apoptosis.

In this study, we have demonstrated that FFA-stimulated macrophage-derived CTSS can induce pyroptosis in vivo and in vitro. We observed that higher levels of FFA promote M1 polarization in macrophage. IRF5 regulates CTSS, which increases levels of trypsinogen and caspase-1 and leads to the posttranslational modification of GSDMD. This activates the cell pyrolysis pathway leading to the deterioration of the pancreas associated with HP. The use of a CTSS inhibitor in a murine model of HP led to less severe disease characteristics and could be a potential therapeutic strategy in patients with HP.

## Conclusions

Here we propose that excess fatty acids induce pro-inflammatory polarization of macrophage and mediate the onset of inflammation and inflammatory death of pancreatic acinar cell. This study denotes the underlying the mechanism of HP and provide a theoretical basis for the development of clinical strategies.

## Supplementary Information

**Additional file 1:** The original blots generated in the Figure 1.

**Additional file 2:** The original blots generated in the Figure 2.

**Additional file 3:** The original blots generated in the Figure 3.

**Additional file 4:** The original blots generated in the Figure 4.

**Additional file 5:** The original blots generated in the Figure 5.

**Additional file 6:** The original blots generated in the Figure 6 and 7.

**Additional file 7:** The sequencing data generated in this study.

## Data Availability

The datasets used and/or analyzed during the current study are available from the corresponding author on reasonable request. The sequencing data generated in this study was shown in Additional files 1 to 7: Supplementary Material.

## Abbreviations

AP: Acute pancreatitis
CTSB: Cathepsin B
FFA: Free fatty acids
GSDMD: Gasdermin D
HP: Hyperlipidemic pancreatitis
IRF5: Interferon regulatory factor 5
PA: Palmitic acid
ROS: Reactive oxygen species
TG: Triglyceride
