# Supplementary figures and images for: Excess fatty acids induce pancreatic acinar cell pyroptosis through macrophage M1 polarization
Source: BMC Gastroenterol. 2022 Feb 19;22:72. doi: 10.1186/s12876-022-02146-8 (PMC8858517; doi:10.1186/s12876-022-02146-8)

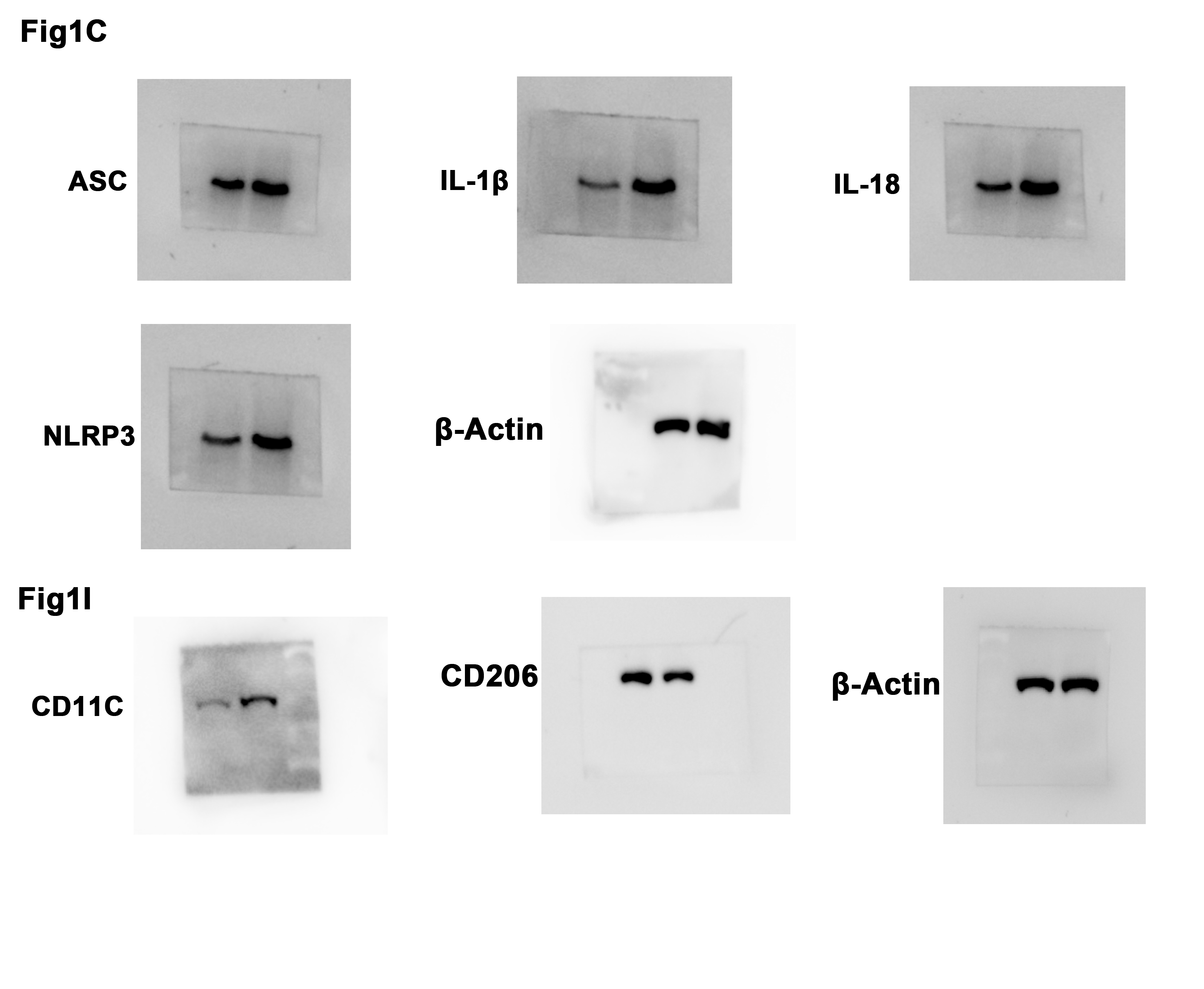

Supplement: Supplementary file 1 — Additional file 1: The original blots generated in the Figure 1. [file 12876_2022_2146_MOESM1_ESM.tif]

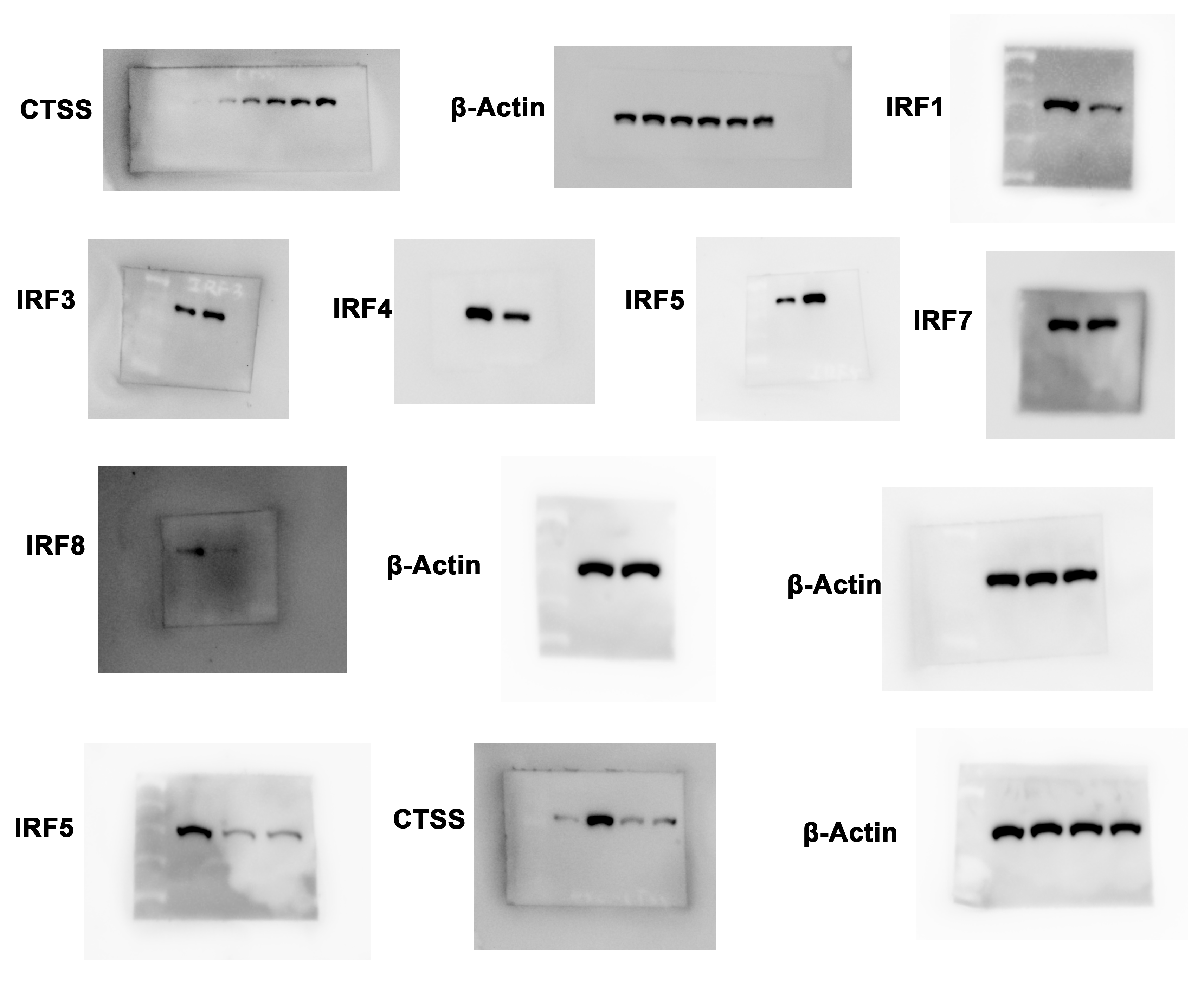

Supplement: Supplementary file 2 — Additional file 2: The original blots generated in the Figure 2. [file 12876_2022_2146_MOESM2_ESM.tif]

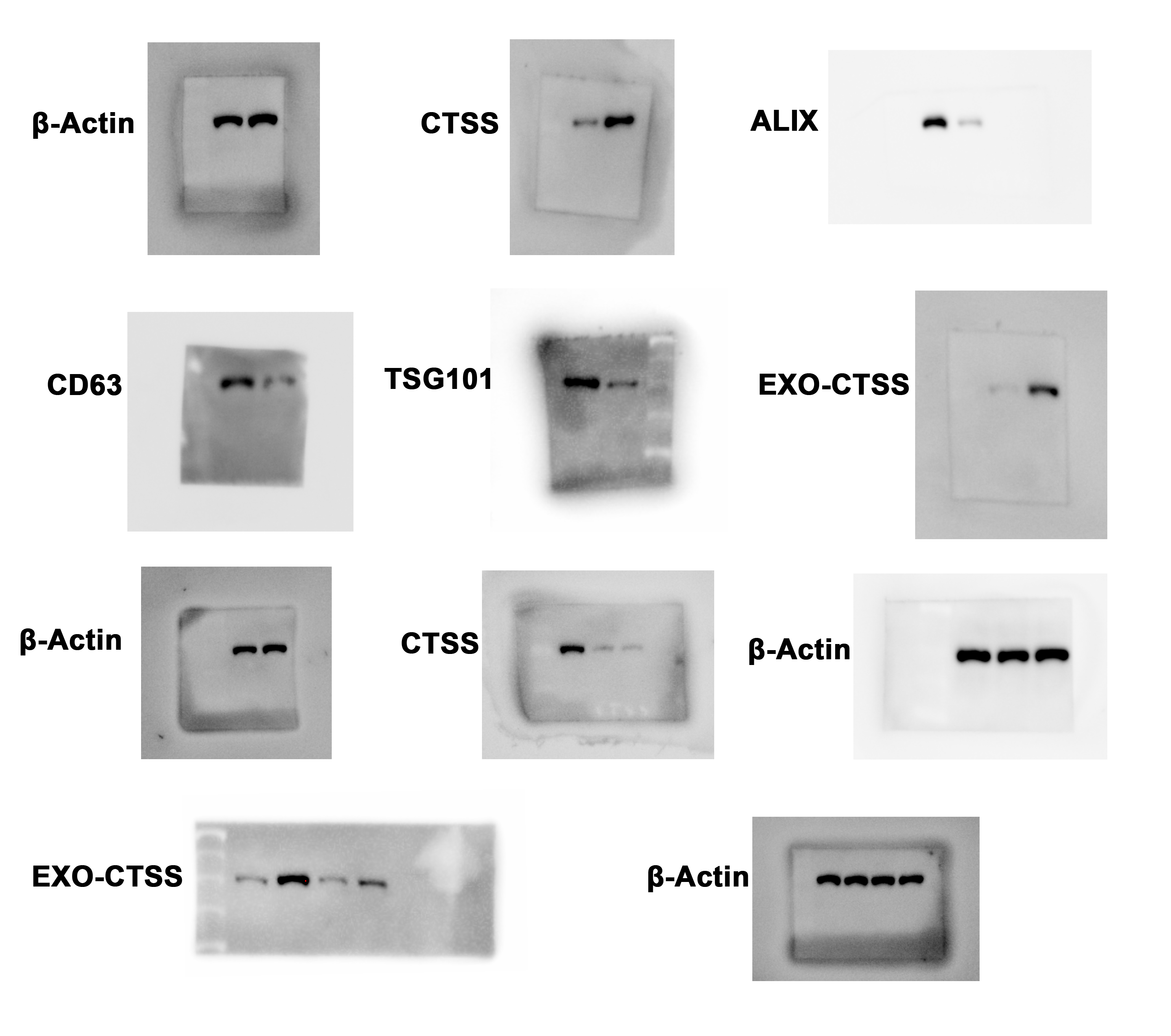

Supplement: Supplementary file 3 — Additional file 3: The original blots generated in the Figure 3. [file 12876_2022_2146_MOESM3_ESM.tif]

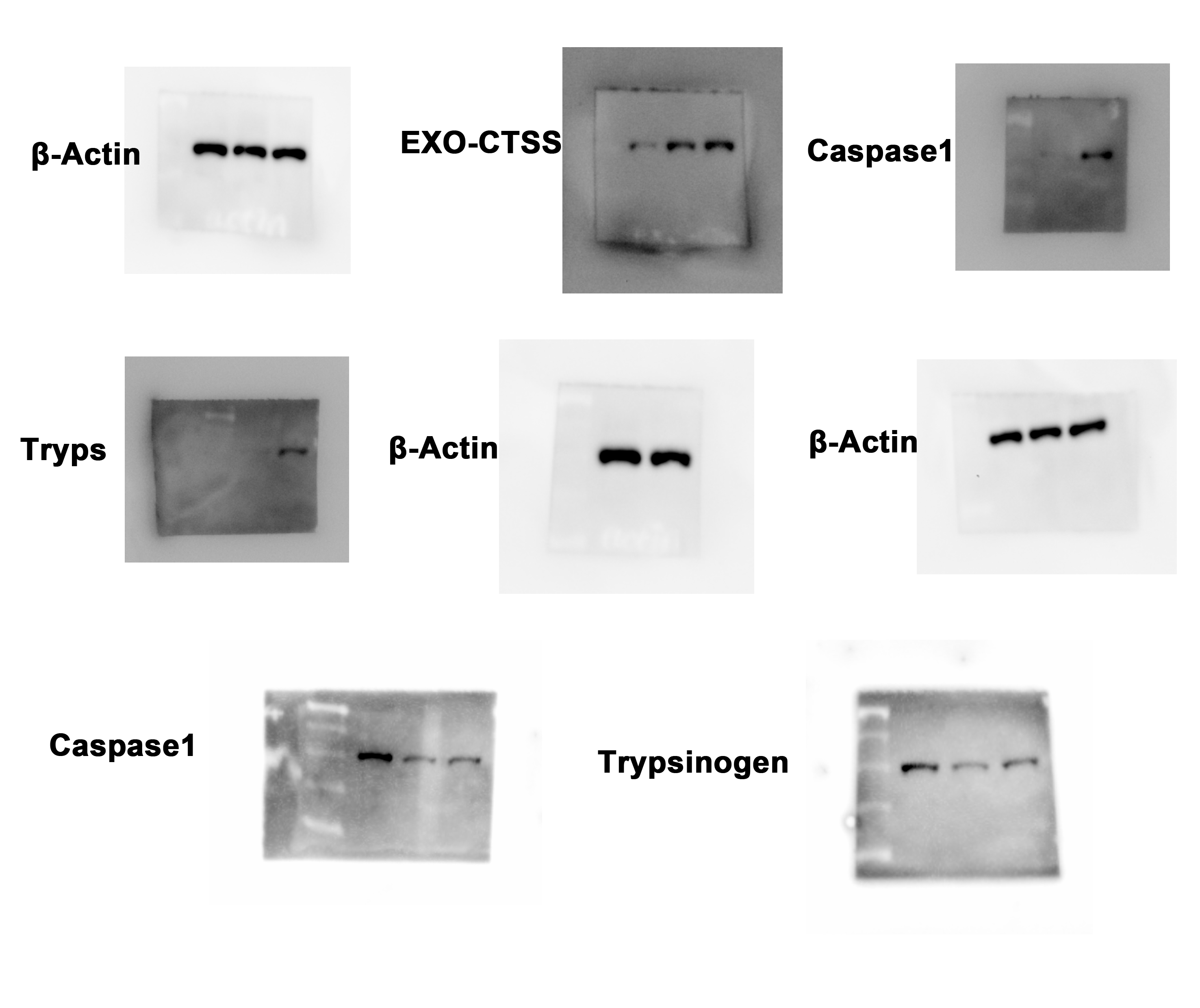

Supplement: Supplementary file 4 — Additional file 4: The original blots generated in the Figure 4. [file 12876_2022_2146_MOESM4_ESM.tif]

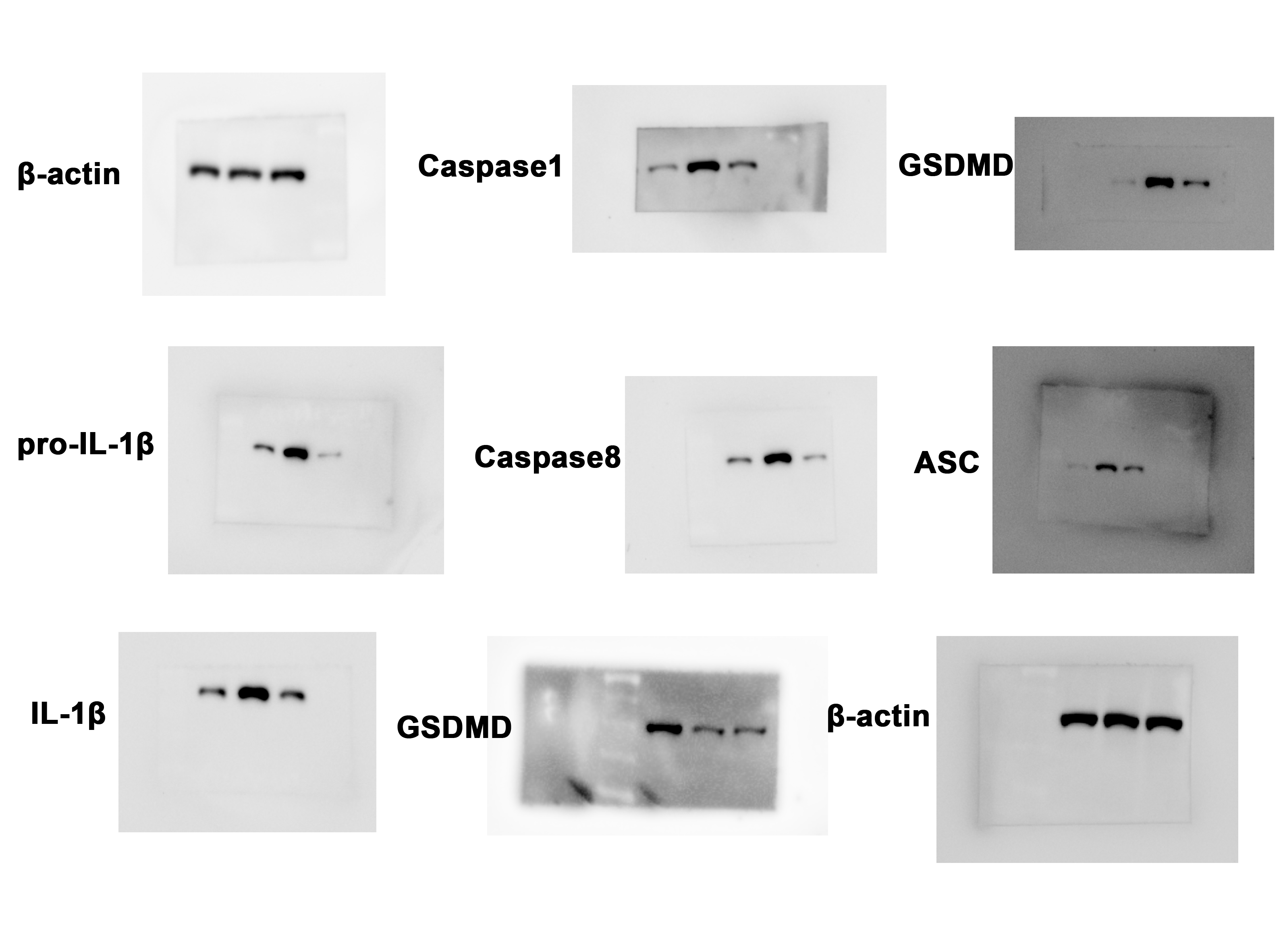

Supplement: Supplementary file 5 — Additional file 5: The original blots generated in the Figure 5. [file 12876_2022_2146_MOESM5_ESM.tif]

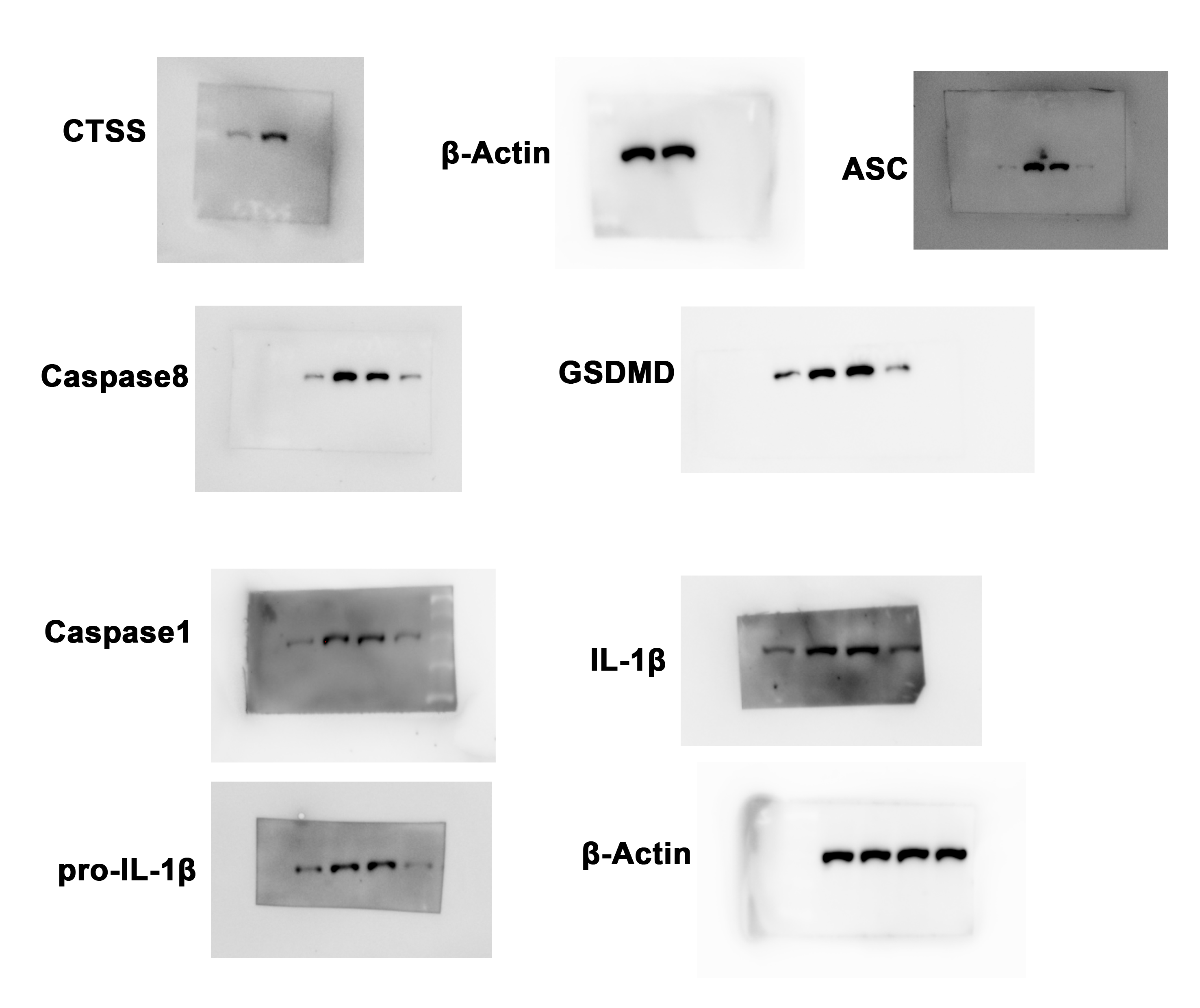

Supplement: Supplementary file 6 — Additional file 6: The original blots generated in the Figure 6 and 7. [file 12876_2022_2146_MOESM6_ESM.tif]
