# Supplementary material for: Excess fatty acids induce pancreatic acinar cell pyroptosis through macrophage M1 polarization
Source: BMC Gastroenterol. 2022 Feb 19;22:72. doi: 10.1186/s12876-022-02146-8 (PMC8858517; doi:10.1186/s12876-022-02146-8)
Supplement: Supplementary file 7 — Additional file 7: The sequencing data generated in this study. [file 12876_2022_2146_MOESM7_ESM.docx]

**Supplementary material for Construction of gene inhibition and overexpression vectors**

CTSS sequencing data：GGAACAGCTATGACCATGATTACGAATTCATGAAACGGCTGGTTTGTGTGCTCTTGGTGTGCTCCTCTGCAGTGGCACAGTTGCATAAAGATCCTACCCTGGATCACCACTGGCATCTCTGGAAGAAAACCTATGGCAAACAATACAAGGAAAAGAATGAAGAAGCAGTACGACGTCTCATCTGGGAAAAGAATCTAAAGTTTGTGATGCTTCACAACCTGGAGCATTCAATGGGAATGCACTCATACGATCTGGGCATGAACCACCTGGGAGACATGACCAGTGAAGAAGTGATGTCTTTGATGAGTTCCCTGAGAGTTCCCAGCCAGTGGCAGAGAAATATCACATATAAGTCAAACCCTAATCGGATATTGCCTGATTCTGTGGACTGGAGAGAGAAAGGGTGTGTTACTGAAGTGAAATATCAAGGTTCTTGTGGTGCTTGCTGGGCTTTCAGTGCTGTGGGGGCCCTGGAAGCACAGCTGAAGCTGAAAACAGGAAAGCTGGTGTCTCTCAGTGCCCAGAACCTGGTGGATTGCTCAACTGAAAAATATGGAAACAAAGGCTGCAATGGTGGCTTCATGACAACGGCTTTCCAGTACATCATTGATAACAAGGGCATCGACTCAGACGCTTCCTATCCCTACAAAGCCATGGATCAGAAATGTCAATATGACTCAAAATATCGTGCTGCCACATGTTCAAAGTACACTGAACTTCCTTATGGCAGAGAAGATGTCCTGAAAGAAGCTGTGGCCAATAAAGGCCCAGTGTCTGTTGGTGTAGATGCGCGTCATCCTTCTTTCTTCCTCTACAGAAGTGGTGTCTACTATGAACCATCCTGTACTCAGAATGTGAATCATGGTGTACTTGTGGTTGGCTATGGTGATCTTAATGGGAAAGAATACTGGCTTGTGAAAAACAGCTGGGGCCACAACTTTGGTGAAGAAGGATATATTCGGATGGCAAGAAATAAAGGAAATCATTGTGGGATTGCTAGCTTTCCCTCTTACCCAGAAATCTAGGGATCCTCTAGAGATATCGTCGACCTGCAG

CTSS raw data：

ATGAAACGGCTGGTTTGTGTGCTCTTGGTGTGCTCCTCTGCAGTGGCACAGTTGCATAAAGATCCTACCCTGGATCACCACTGGCATCTCTGGAAGAAAACCTATGGCAAACAATACAAGGAAAAGAATGAAGAAGCAGTACGACGTCTCATCTGGGAAAAGAATCTAAAGTTTGTGATGCTTCACAACCTGGAGCATTCAATGGGAATGCACTCATACGATCTGGGCATGAACCACCTGGGAGACATGACCAGTGAAGAAGTGATGTCTTTGATGAGTTCCCTGAGAGTTCCCAGCCAGTGGCAGAGAAATATCACATATAAGTCAAACCCTAATCGGATATTGCCTGATTCTGTGGACTGGAGAGAGAAAGGGTGTGTTACTGAAGTGAAATATCAAGGTTCTTGTGGTGCTTGCTGGGCTTTCAGTGCTGTGGGGGCCCTGGAAGCACAGCTGAAGCTGAAAACAGGAAAGCTGGTGTCTCTCAGTGCCCAGAACCTGGTGGATTGCTCAACTGAAAAATATGGAAACAAAGGCTGCAATGGTGGCTTCATGACAACGGCTTTCCAGTACATCATTGATAACAAGGGCATCGACTCAGACGCTTCCTATCCCTACAAAGCCATGGATCAGAAATGTCAATATGACTCAAAATATCGTGCTGCCACATGTTCAAAGTACACTGAACTTCCTTATGGCAGAGAAGATGTCCTGAAAGAAGCTGTGGCCAATAAAGGCCCAGTGTCTGTTGGTGTAGATGCGCGTCATCCTTCTTTCTTCCTCTACAGAAGTGGTGTCTACTATGAACCATCCTGTACTCAGAATGTGAATCATGGTGTACTTGTGGTTGGCTATGGTGATCTTAATGGGAAAGAATACTGGCTTGTGAAAAACAGCTGGGGCCACAACTTTGGTGAAGAAGGATATATTCGGATGGCAAGAAATAAAGGAAATCATTGTGGGATTGCTAGCTTTCCCTCTTACCCAGAAATCTAG

sh-CTSS #1 sequence:

5'- CACCGCCAGTGGCAGAGAAATATCATTCAAGACGTGATATTTCTCTGCCACTGGC-3'

sh-CTSS #2 sequence:

5'- CACCGGCTTCATGACAACGGCTTTCTTCAAGACGGAAAGCCGTTGTCATGAAGCC-3'
